# Lectures on Subjects Connected with Clinical Medicine; Comprising Diseases of the Heart

**Published:** 1847-06

**Authors:** 


ART. 3. *Lectures on subjects connected with Clinieal Medicine; comprising Diseases of the Heart.* By P. M. LATHAM, M. D., *Fellow of the Royal College of Physicians; Physician extraordinary to the Queen &c.* Philadelphia Ed. Barrington, and Geo. D. Haswell. 1847. 8vo. pp. 365.

We have already made this work the subject of a brief notice, [see No. XI, Vol. II, April,] at that time we had not bestowed upon it careful study. The reputation of the author as a practical observer and teacher, together with the prepossessions derived from a cursory examination of the volume made us desirous to avail ourselves of our earliest moments of leisure, to give it an attentive and thorough perusal. Our expectations have not been disappointed, it is a valuable contribution to the literature of Practical Medicine, presenting as it does, the leading facts pertaining to the diagnosis of cardic diseases with admirable perspicuity and abounding in valuable observations which are often of wider applicability, than to the particular affections of which it specially treats. The work, as its tittle denotes, consists of lectures, which were delivered at St. Bartholomew's Hospital, several years previously to their present publication. The colloquial style, in which one instruction is most agreeably and effectively communicated, is preserved—a quality which will render it more attractive to the reader, although, being necessarily more discursive than a systematic treatise, it is somewhat less conveinent for after reference to particular topics. The work bears internal evidence of candour and sincerity, and of a desire to tell the truth, the whole truth, and nothing but the truth. These are recommendations which it is the more important to refer to, in as much as it is unfortunately to be confessed, that they are not of universal application to the works of Medical Teachers. The various departments of Practical Medicine afford such a field for the self-deceptions of excessive enthusiasm, and so many temptations to egotistical assumptions, that we are forced to inquire, before receiving with confidence what purports to be the fruits of individual investigation and experience, whether their value be not impaired by a deficiency of good sense, or too little of that perfect uprightness of mind which regards truth as the paramount object in scientific researches. We feel assured that the readers of Dr. Latham's lectures, will concur with us in the conviction, that they display, not only extensive opportunities for observation diligently improved, and an accumen above the common order ; but, what is as much, if not greater praise, that they evince a degree of prudence, judgment, and honesty of intention which are essential to render instruction reliable and useful.

With such views of the work it is but due to it, and to our reader, to present some of the more important practical precepts which it contains ; and we purpose in this and a preceeding number, to devote to an analytical review, as much space as is consistent with a proper proportion of other matter, submitting, in connection with the different topics which it involves, such comments as may suggest themselves.

We are led to review the work, also, not alone from its intrinsic merits, but from the claims of the subject, which, we fear, are by no means fully appreciated by the mass of Practioners in our country. The diseases of the heart are too often regarded as of but little account to the general practitioner. He looks upon them as constituting a melancholy class of lesions, of which but little precise knowledge can be acquired, anterior to autopsical examinations ; and what little is attainable prior to the death of the patient, in the way of diagnosis, is deemed of small practical importance. The study appears to him, on a superficial view, to be pursued except as a specialty, and he therefore is reluctant to enter upon it. This impression is as erroneous, and unjust to the subject, as it is prevalent. The application of physical exploration to cordial affections, aided by modern researches which have elucidated the correct physiology of the heart's action, has given to their diagnosis even greater simplicity and precision, than belong to the study of pulmonary diseases ; in fact, the principles involved in the differential diagnosis of the different forms and localities of organic lesions of the heart, are, in the main as readily apprehended, as they are easy and satisfactory in the application. There are no difficulties in the pursuit which should repel the student, or practioner, from engaging in it with the utmost confidence in his ability, to secure, by his own exertion, aided by books alone, if oral instructions are not available, most of its practical advantages.

The important relation, too, of the diseases of the heart, are worthy greater than are generally supposed. The cases in which death follows directly from lesions of this organ are few, in comparison with those in which this event is traceable to the same source through indirect influences. It is now well ascertained that a host of secondary diseases are attributable to affections of the heart often when the latter are not prominent, being of course overlooked by those who do not take into account the connections existing between them and the disorders, which, from their prominence, arrest and limit the attention. But of this, and other matters connected with the all-important subjects, we may perhaps take occasion to offer farther remarks, as we pass in review the topies treated of in the work before us.

The first four lectures of Dr. Latham's work, are occupied with an exposition of the natural sounds, impulses and resonances of the heart ; the deviations from healthy sounds or murmurs, and the principles of auscultation as applied to this organ. The facts and rules enunciated in these lectures are those, in the main, which are more comprehensively and elaborately treated of in the incomparable work of Hope. We cannot supply an epitome of them but must content ourselves with advising those of our readers who may be wholly unacquainted with this department of diagnosis, to consult the latter work, as well as that under present notice. As regards the adventitions and modified sounds or murmurs, they are all either produced from causes existing within the cavities of the organ by the current of the blood through the orifices; or, on the other hand externally, by friction of the outer surface of heart upon the inner surface of the pericardium, one or both surfaces being altered by disease, so that the motion of the organ no longer takes place smoothly and noiselessly. Dr. L. distinguishes these, two classes of murmurs by the titles of *Endo-cardial*, applied to the former; and *Exo-cardial*, to the latter. These new terms are well enough, yet there was not much occasion for them. The Endo-cardial murmurs are but varities of what is commonly known as the bellows murmur, and the exo-cardial are the attrition murmurs. The bellows murmur, in by far the greater proportion of cases in which it is discoverable, is to be regarded as unequivocal evidence of valvular disease. The exceptional instances are those in which it occurs as a symptom of diminished and impoverished blood in anœmia. These cases are generally discriminated without difficulty. They are marked by the various and distinctive evidences of the anœmia state, and the murmur is less constant, and invariably of a soft character, never presenting any of the various degrees of roughness which have been named by different writers, sawing, rasping, filing, &c., after the analogies which these terms express. These varieties, it may be observed, which, at first, seem to puzzle the beginner, possess no special significancy except as denoting the mere fact of valvular disease. They give little or no information as respects the location or the character of the lesions.

It is an interesting and not unimportant point in diagnosis to determine the particular orifice or orifices in which the bellows murmur is produced in particular cases ; on this point it is well known to those familiar with the writings of Dr. Hope, that a few, simple practical principles are therein laid down, which, Dr. H. states, may be relied upon with almost certain confidence. To illustrate the facility of their application, the author declares, that after explaining these principles to several pupils, by means of diagrams, devoting to it only fifteen minutes, and then introducing them to several patients with well marked different forms of valvular diseases, they were able to diagnosticate with correctness, for the most part, although they were previously, entirely unacquainted with auscultation as applied to cardiac diseases! These principles involve the relations of the murmurs to the natural sounds of the heart, considered in connection with the causes which produce these sounds ; the locality, at which the murmurs present a percussion of intensity, and the direction in which they are most readily transmitted ; the timbre or pitch of tone, which is ingeniously represented by whispered words and letters, &c. To explain these points in this connection would be to elucidate the whole subject, which it is not our purpose to attempt to do. We must again refer the reader to the work of Dr. Hope, as well as to that before us. We would only say to the novitiate, do not imagine that the bellows murmur is a sign, which it is a formidable undertaking to attempt to grasp by the sense of audition; first, learn to disconnect the two sounds of the heart—to appreciate the *tic-tac ;* and the first case which presents itself in which the bellows murmur exists, you will be sure to recognize it, and if you will adopt the habit of listening to the sounds of the heart in aged persons, whether they have symptoms of heart disease, or not, you will not wait long before you will meet with sufficiently palpable illustrations.

With regard to the practicability of locating the valvular lesion from the several circumstances mentioned, Dr. Latham does not speak quite so confidently as Dr. Hope. He asks "are these facts true, and stable, and general enough, to hold the place of principles ? and if they be, will they bear to be taken, always, and without reserve, as the sure exponents of endo-cardial murmurs, so as to fix the valve or orifice from which they proceed ?" He answers, "I dare not affirm so much, I do indeed still make use of them as principles, but I am less peremptory about the certainty of their application I than was a year or two ago; often the event has been just as they would indicate, but occasionally, it has been contrariwise ; and the exceptions I have not always been able to explain, without prejudice to the assumed principles." He adds. "But we should not be in a hurry to abandon such general facts as these, which have often led us right, because they have sometimes seemed to lead us wrong. We should rather suspect the occasional interference of counteracting circumstances, which we do not yet understand." The last clause of the quotation expresses, probably, the true aspect in which cases of an apparently exceptional character should be regarded. The fact equally obtains in the application of principles of physical diagnosis to pulmonary diseases, and with respect to similar liabilities to error in the latter case, the same philosophy, holds good. In neither instance are we to undervalue or neglect means which almost always are capable of affording certain and valuable information, because, in a few cases, owing to causes which our knowledge does not, as yet enable us fully to comprehend, the inferences which we draw from the use of those means of information prove to be erroneous. Yet this is not unfrequently advanced as a sufficient argument against the validity and usefulness of physical diagnosis. Admit it to be a logical argument, and apply it to diagnosis from rational or vital symptoms, and where would it lead us? Is there a single symptom or group of symptoms of the latter class, which could bear a test so rigid as this. If it be insisted that in order for diagnostic signs to be worthy of any confidence, they must posess and maintain invariable significancy in every instance there would soon be an end to semeiology as a branch of Medical Sience.

In reading the first four lectures, devoted, as we have said, to the normal and abnormal sounds of the heart, our attention was arrested by one source of deception, which we do not recollect to have been mentioned by other writes, and it is one which the practitioner should be apprised of. We allude to the practibility of producing the bellows murmur by pressing down the ribs too firmly with the ear, or stethescope. Dr. L. gives the following case illustrating this point.

"A little boy, aged eight years and a half, high-spirited and vivacious, but thin and out of health, was brought to me under a suspicion of disease of the heart. Its impulse was not felt beyond the apex, but *there* it was in excess; yet there was no larger space of dulness than natural in the præcordial region. Upon auscultation, however, this remarkable peculiarity was made out. When the ear or the stethoscope rested gently upon the præcordial region, no unnatural sound whatever was heard. But when either the ear or the stethoscope was applied with such force as to cause the ribs to sink a little below their natural level, then a loud bellows-murmur sprang up. The space at which it was heard, and not beyond it, was just so far as the mouth of the stethoscope covered, when it was placed upon the cartilage of the third rib as a centre. Below and above this spot the murmur vanished, and it was audible neither in the course of the aorta nor in the carotids."

"This case, which occured to me five years ago, has made me watchful ever since, lest haply I might sometimes create the murmur I was in search of. And it is no needless caution where the patient is young and the frame-work of the chest is yielding. Never, indeed, the chest being not deformed, never but this single instance have I produced a murmur simulating that of valvular disease. But very often, when, over-earnest in what I was about, I have pressed too heavily upon the præcordial region, a sort of jarring sound has reached my ear, and brought with it the suspicion of disease, until setting the heart free from the weight and the restraint which I had inadvertenly imposed upon it, I have at once lost the sounds and the apprehensions too, which had arisen from my own awkward manœuvering."

"It is well to know this possible fallacy of our own making, and so to guard against it."

Twenty lectures following the four already referred to, are occupied with the discussion of inflammation affecting the pericardium and endo-cardium; The connection of these inflammatory affections with rheumatism; the treatment of rheumatism with reference to them, as well as the special treatment which they require; the lesions which result from them, and the ulterior consequences flowing from the latter. We will proceed to select from the portion of the work occupied with these topics such practical considerations as shall appear to us most interesting and important, being guided, of course, by the necessity of restricting this article within reasonable limits.

The very frequent complication of Endo-carditis, with or without pcrcarditis, in cases of acute rheumatism is one of the fixed facts of modern science. Dr. Latham was among the first to investigate the connection which subsists between these affections; a connection now sufficiently established and of vast practical import. The following extract contains an account of the rise and progress of this discovery, as it may properly be entitled, in so far as the observations of British Pathologists are especially concerned. We regret to see some evidences in the quotation of a disposition, which is so common among British writers, to disparage or overlook the claims of their gothic neighbors and scientific researches. The name of M. Bouillaud is identified with the modern history of endocarditis, and its connection with rheumatism; yet our author refers to him only in a way admitting of a construction which contains rather an equivocal compliment. He attributes to him the credit of having first determined the frequent exisience of the endocardial complication by directions, but this was owing to the fact that M. Bouillaud's experience furnished fatal cases, while that of the author presented no opportunities for post-mortem examinations! We may be entirely at fault in thinking that any reflections were intended by the manner in which the subject is refered to, but we cannot repress such a suspicion, more especially as M. Bouillaud's name is not otherwise mentioned in the passage, which, in itself, is an act of negative injustice. If we are correct in our surmise, the American reader will at least think with us, that a demonstration of personal or national prejudice is not less contemptible in Dr. L., than in any ofther writer. We repeat, that we are sorry to observe such a desire for criticism in a work which generally exhibits a spirit of firmness and candor worthy all praise. But to the extract :—

"Endocarditis is at present chiefly known as a concomitant of Acute Rheumatism."

"In the year 1826 I was the first to teach the students of this hospital the fact, that whenever the heart was affected in acute rheumatism, a sound different from the sound of health always accompanied its contradiction. This was then a new fact, and one of immense importance; and all succeeding observation has gone to confirm its truth. This sound in the vast majority of instances was the bellows' murmur. I must not call it endocardial, for it was not yet known to be so. But in some, instead of the bellows' murmer, it was some strange sound difficult to describe; and in others this strange indescribable sound and the bellows' murmur seemed to occur together: there was a mixture of both.

"My notion then was, that all these sounds arose in some way or other out of inflammation of the pericardium; and taking them all, several and in combination, as the signs of pericarditis, I was amazed to find how far the frequency of its occurrence in acute rheumatism exceeded the common calculation and beliief."

"In process of time I found, upon a comparison of cases, that where in acute rheumatism and bellows' murmur ocurred alone, the affection of the heart was upon the whole far less severe and far less perilous to life, than where some other unnatural sound occurred alone or in combination with it. I observed, too, that in some cases where the bellows' murmur was unequivocal, the patient betrayed no uneasiness, no palpitation, in short, no other symptom which could give the least suspicion of a diseased heart; yet that in the great majority of instances where it once existed it remained permanent as long as the patient continued under my care and observation."

"At length I began to doubt whether the bellows' murmur arising in the course of acute rheumatism was really derived from the pericardium, and to suspect that it proceeded from the internal lining. But for years the practice of this great hospital did not afford me a single opportunity of resolving my doubt, or of confirming my conjecture. For of that disease of the heart, which, coming on during acute rheumatism, is characterised by the bellows' murmur no patient of mine ever died, and I could learn nothing about it from dissection. But what my own experience would not furnish, M. Bouilaud's has supplied. Many have died during the active progress of this disease under his care, and dissection has found it to be inflammation of the endocardium. Thus we are indebted to M. Bouilaud for our first knowledge of this important fact,

Nearly about the same time Dr. Watson and Dr. Stokes of Dublin, further illustrated this important subject by separating those other sounds of the heart, which, I have mentioned to occur in acute rheumatism, from the bellows' murmur, and analysing them apart. These they found to possess the character of attrition, as if produced by surfaces moving to and fro upon each other, and traced them home to their local origin in the pericardium, and showed the condition of their production to be a defective lubricity or a ruggedness and unevenness of that membrane, such as would result from inflammation. In short, they showed these to be the proper signs of pericarditis, as the bellows' murmer is of endocarditis ; that when in acute rheumatism either occur alone (as it often does), the disease is simple pericarditis, or simple endocarditis ; and that when in acute rheumatism both occur together (as they often do), there is a mixture of the two diseases in the same subject."

"The bellows' murmur coming on in the course of acute rheumatism is a sure sign of inflammation of the endocardium. Here, then. we will drop this exceedingly vulgar name, and call it "endocardial" again. But observe, it is the general character of the morbid actions predominant in the system at large which determines the particular character of the local disease out of which the endocardial murmur arises. They are inflammatory, and it is inflammation.

"In endocarditis, besides the endocardial murmur, there may be other symptoms present directly referable to the heart, or there may not. There may or may not be pain. There may or may not be an excessive impulse, or an intermittent, irregular, or fluttering action of the heart. But the fact of endocarditis is not rendered more or less certain by their presence or absence."

"There may be both pain and palpitation ; yet endocarditis cannot be surely inferred to exist, unless there be the endocardial murmur withal.* There may be neither pain nor palpitation, yet endocarditis cannot be inferred not to exist, if the endocardial murmur alone be present."

"Seeing, then, that the endocardial murmur alone can determine the existence of endocarditis, you are required to search after it in every case of acute rheumatism. I say emphatically *to search after it*, because it is one of those signs which must always be sought before it can be found. It does not intrude itself upon our notice like palpitation, or an irregular pulse. The patient does not draw our attention to it as he does to pain. The physician must make it out entirely for himself. And indeed it is infinitely important that he should have the earliest possible notice of it with a view to the earliest possible application of the remedy."

"Never omit, therefore, to listen to the præcordial region whenever you visit a case of acute rheumatism, and visit a case of acute rheumatism oftener perhaps than you otherwise would do merely for the sake of so listening. All may seem well, and to be going on well. The general symptoms may be far from severe. The chest may be free from pain. The heart's action may not awaken suspicion by its force or irregularity. Nevertheless, its internal lining may be inflamed, and, if you listen, the endocardial murmur may convey the momentous fact directly to your ear."

The practical precepts contained in the foregoing quotation cannot be too impressively commended to the attention and recollection of the practitioner.

Pericarditis, like endocarditis, in the experience of Dr. L., originates in the vast majority of instances as a complication of acute rheumatism. On this point he holds the following language:—

"My own experience of pericarditis is mainly derived from what it is, as an accompaniment of acute rheumatism. I have seen the disease, indeed, under other circumstances. But it has been very seldom; so seldom indeed, that I have little acquaintance with other conditions external or internal conducting to it. I can neither tell whence to look for it nor when to expect it, except when it occurs as a part of acute rheumatism."

"The pericarditis, which is acute and rapidly progressive, and, unless arrested by timely and effectual treatment, full of peril, this is the pericarditis I mean. And with this the practice of a large hospital has rendered me familiar by presenting me every year with numerous instances of it in alliance with acute rheumatism. But separate from acute rheumatism, even the practice of a large hospital does not present me with more than an instance or two of it in several years. And, as of the disease itself, so of all the

* I do not say that it would not be fairly suspected, and that it would not be right to act as decidedly upon the suspicion in such a case as upon the matter of fact. It certainly would.

symptoms and auscultatory signs by which I learn its existence and direct its treatment, my chief knowledge comes from acute rheumatism."

The occurrence of pericarditis in connection with rheumatism, is by no means a fact of such recent discovery as that of the complication of endocarditis. But until the application of physical diagnosis, the former, in a large proportion of cases in which it co-existed, was overlooked, inasmuch as the rational evidences of cardiac trouble—such as pain, irregular action, &c., are frequently not prominent enough to arrest the attention of either the patient or physician. The complication of both forms of disease, separately or combined, was seldom recognized at an early stage; and, hence arose the idea that the heart affection, when it was discovered, was a *metastasis*, rather than a *complication*. It was in fact, only ascertained to exist until it proceeded so far as to occasion prominent symptoms from the amount of injury which had imperceptibly taken place, and when the opportunity for successful practice had in a great measure passed by unimproved. Hence, the immense practical importance of the results of late investigations.

The most unequivocal evidence of the existence of pericarditis is afforded by the presence of the attrition, or, as Dr. Latham prefers to call them, the endocardial murmurs. If these murmurs are present, the diagnosis is thereby rendered easy and precise. A common opinion is, that, in a large proportion of cases, after considerable effusion has ensued, the attrition murmurs disappear, to return perhaps again, when absorption has diminished the serum, bringing the roughened surfaces again into contact. Dr. L., however, entertains the belief, founded on his experience, which must have been extensive, that effusion, although considerable, does not abolish the murmurs produced by friction. We cannot but fear that the cases are not few in which this valuable sign is not available for diagnosis, but our apprehensions are derived rather from a rational view of the subject than from experimental knowledge. We sincerely hope it may prove as Dr. L., teaches. After speaking of dulness on percussion over the præcordial region as an important diagnostic sign, he proceeds to say, as follows:—

"But do these two, viz., the murmur and the dulness, bear the same relation to each other as signs of disease within the pericardium, as they have been seen to bear as signs of disease within the pleura? In pleurisy the attrition-sound and the dulness are never coincident, but are alway found to supersede each other, one ceasing as soon as the other arises. Is this the case in pericarditis? My own experience would answer almost absolutely, "No!" As soon as I have discovered the exocardial murmer at any part of the præcordial region, so soon have I almost always found dulness to percussion. And, to whatever extent the dulness to percussion has spread beyond the præcordial region, the murmur has accompanied it, even as high as the first left rib, and beneath the sternum, and far beyond it, even to the juncture of the cartilages with the right ribs. Further, I have known dulness of the præcordial region to be the *first* sign, and to subsist several days *alone*, and yet the attrition-sound has at length been superadded to it, when they have thenceforth continued together. In pericarditis, then, this I take to be the general truth, namely, that the murmur, which is produced by lymph deposited upon the surfaces of the membrane, is neither abated, nor abolishod, nor otherwise altered in its character by the serum effused within its cavity. It is not, however, the *universal* truth."

Indeed, I have seen a few instances of pericarditis, where the murmur, clearly heard day after day, has at once become very indistinct, or has been suddenly lost together. The disease has been at its greatest activity, and the heart at the very time has suffered some extraordinary bafflement of its actions. But the sound, thus abated or abolished, has suddenly returned, and in a day or two has been as perfect an exocardial murmur as it was before, and then the heart's actions have recovered their regularity In these instances the remedy which has thus instantly relieved the heart and restored the sound has been a large blister vesicating the whole præcordial region, and a wide space around it."

"Now dwell a little thoughtfully upon these instances, and upon the several attendant circumstances, both when the sound goes and when it returns, and above all, upon *the remedy*, and its immediate effect; and then say what they can denote, but now a sudden increase, and now a sudden decrease, of fluid within the pericarpium?"

"But still, if the common experience be in accordance with my own, and if my own speak the truth in this matter, the fact will be that serous effusion within the pleura *always* obliterates the attrition-sound and that serous effusion within the pericardium *generally* leaves it unaltered."

The following passages refer to physical signs, which, when present, are, of course, valuable indications:—

"In pericaditis, while the præcordial region is dull to percussion and the exocardial murmur is heard, an undulating motion often becomes visible to the eye in some of the spaces between the cartilages of the second and third ribs, or of the third and the fourth, or between both at the same time, that I have seen this motion, and never in any other situation."

"So, too, in pericarditis, while the præcordial region is dull to per cussion and the murmur is heard, a vibratory motion often becomes sensible to the touch in some of the spaces between the cartilages of the ribs on the left side. As I never saw the undulatory, so I never felt the vibratory, motion elsewhere than either between the cartilages of the second and third, or of the third and fouth ribs, or between the cartilages of both simultaneously."

"The vibration (I believe) is the more frequent of the two, and often occurs unaccompanied by any visible undulation. But the undulation was never apparent to my eye without my finger being able to detect a sensible vibration at the very same spot."

The presence of peri—and endocarditis, occuring separately or together, as accompaniments of acute rheumatism, is made by Dr. L., the subject of numerical investigation. The following are the more important statistics which he presents: Out of 136 patients occurring at St. Bartholemew's Hospital, between the year of 1836 and 1840 both inclusive, 75 being males and 61 females, the heart was exempt from disease in 46 and affected in 90.

The seat of the disease in the heart was in the endocardium alone, in 63; in the pericaadium alone, in 7; in the endocardium and pericardium, in 11; and doubtful in 9 cases. The deaths were 3 in number, in each of which both the endocardium and pericardium were affected.

We quote his own comments on this numerical exposition:—

"Here are momentous facts which go (I suspect) a good deal beyond the ordinary notions entertained by medical men of this matter. It is believed that among the sufferers of acute rheumatism, an individual now and then unluckily has his heart inflamed. The thing is looked upon as an accident which, if not very rare, yet is not very common. But it appears, from the event, not of a dozen or twenty cases merely, but of a number large enougir to furnish the measure of what naturally belongs to the disease, that as many as two-thirds of those who have acute rheumatism also suffer inflammation of the heart."

The small ratio of fatality may seem to give an erroneous impression concerning the real danger of the complication. Out of the whole number (136) three only died. In each of the fatal cases the endocardium and pericardium were both involved. Not one of the 63 cases in which the endocardium alone was affected proved fatal. Is it to be therefore inferred that endocarditis is not a serious disease? Let us follow Dr. L's investigation somewhat farther:

"But of these 63, whom the endocarditis did not kill, and who, as far as general symptoms could not be trusted, might be pronounced convalescent or well, auscultation still told us that, after the inflammation had ceased, the membrane recovered its complete integrity of structure only in 16, and that it remained permanently injured in 46. For of the 30 males, the subjects of rheumatic endocarditis, the endocardial murmur ceased entirely only in 8; while it remained, after they were convalescent, and as long as they continued under observation, in 22. And of the 33 females the endocardial murmur ceased entirely only in 9; where it remained in 24."

"Thus, while inflammation arrested and life saved in all the cases which occurred, even 63 in number, do indeed sufficiently testify how small is the present peril of life from rheumatic endocarditis, yet the entire restoration of the endocardium to its perfect structure in 17 only, and the permanent injury done to it in 46, denote a most fearful disease in regard to its distant results. For the probability is as great as four to one, that inflammation befalling the endocardium will become the rudiment of disorganization to the entire heart."

The danger, then, is, not of a fatal result proceeding directly from this complication, but of subsequent organic lesions, which will sooner or later, in the large proportion of cases, eventuate in death. Here it is that the vast practical importance of these investigations lies:—organic lesions within the heart's cavities in most instances are due to pre-existing endocarditis; and endocarditis, in most instances, occurs in connection with rcute rheumatism. The practical considerations belonging to these relations, which late investigations have established, are too obvious to require comments.

The ulterior consequences of pericarditis, as a single complication, are less serious. The reader will note that the ratio of the cases in which this complication existed alone is considerably less than of those in which endocarditis was present alone, or endocarditis and pericarditis conjointly. Of the 7 in the statistics which have been given none died; nor in a single case, did there continue any attrition or exocardial murmur, or other indications that the organ had not resumed the conditions of health.

The facts presented in the foregoing analysis are certainly of a highly interesting character, and possess a degree of importance which is not improperly characterised by the author as *momentous*.

Dr. L., next takes up the subject of "*inflammation of the lungs accompanying acute rheumatism, either alone, or in combination with endocarditis, or with pericarditis, or with both.*"

We shall content ourself with adducing simply his statistics on this subject, with a single remark or two. Inflammation of the lungs was found in 24 of the 136 cases. These 24 cases were made up of 4 cases of bronchitis, 18 of pneumonitis, and 2 of pleuritis. Of the 46 cases in which the heart was unaffected, the lungs were inflamed in 5; of the 90 cases in which the heart was inflamed, the lungs were inflamed in 19; of the 63 cases of endocarditis, the lungs were inflamed in 7; of the 7 cases of pericarditis the lungs were inflamed in4; of the 11 cases of endocarditis and pericarditis simultaneously, the lungs were inflamed in 8.

It is obvious that the collection of cases is not large enough for any positive deductions. In so far as inferences are admissible, they are that the "probability of inflammation of the lungs arising out of acute rheumatism is small; and that the probability is not at all augmented by its alliance with endocarditis;" that it oftenest associated with pericarditis when the latter disease of the heart exists alone, and that it occurs next in greater proportion in cases of pericarditis and endocarditis combined.

We come now to the therapeutical indications involved in the diseases under consideration, and since, it sufficiently appears from the foregoing facts, that heart affections are most intimately connected with the treatment of acute rheumatism, Dr. L., in the first place, considers this topic. It is well known that various and quite opposite methods of treating rheumatism are advocated by different practitioners, each method claiming to be sustained by satisfactory experimental results. Among the most prominent of the methods referred to, are bleeding, and mercurial purgation. Admitting that each of these plans, pursued singly, will apparently succeed, Dr. L., does not deem it a legitimate inference that the cure is effected by the powers of nature, independently, or in spite of the peculiar methods of treatment. He thinks that their efficacy admits of being reconciled, without affording any occasion for disparagement of medical science. Rheumatism offers several indications. One of these indications arises from morbid fulness of the vessels, the increased activity of the circulation, and the increase in the fibrinous constituents of the blood; this indication calls for blood letting, which, within certain limits, will be useful, but may be carried too far. Another indication is furnished by the pain and other disorders of the nervous system. This class of symptoms is appropriately met with opiates. This plan Dr. L., has pursued formerly to a considerable extent, and is satisfied that it exerts a decided efficacy in relieving the suffering, and shortening the duration of the disease. To be efficient, opium must be given in sufficient quantities to *subdue* the severity of the symtoms. Lastly, the secretions are manifestly disordered; and hence, purging by calomel may be rationally supposed, and is practically demonstrated to accomplish useful purposes. Thus it is, that each plan, although different from, and in a measure opposed to the others, secures its own objects, which are useful in so far as they go. Each is addressed to a single class of symptoms, or to distinct elements of the disease, and each possesses a certain measure of utility corresponding to the relative importance of the limited purposes which it subserves. With respect to the several methods mentioned, the author presents many valuable practical considerations to which we could not do justice by any condensed analysis.

It follows as a conclusion from the general view taken of the different kinds of treatment recommended, that the true policy is to make them all available by combining them together. This is the plan advocated. He says:—

"But there is a plan of treating acute rheumatism which is juster and safer, and applicable to more cases, and more successful than any of them. And that plan is a compound of all three.

"This compound method, while it works with all the means which have recommended, stops short of what is harsh and excessive in their use, and yet compasses with more certainty the successful result."

"For I believe, that in the treatment of this disease, and in the same cases, by the judicious use of opium you may spare blood, and by the judicious use of bleeding you may spare opium; that by calomel and purgatives properly administered, you may make bleeding and opium less needful, and that by bleeding and opium discreetly employed you may leave less to be effected by calomel and purgatives."

"Hitherto you have seen how, in the management of acute rheumatism, you may deal with blood-vessels, and with the nerves, and with secreting organs separately, and with what effect. Presently you will see how and with what effect you may deal with them simultaneously; and how your different remedies once set a-foot, and pursuing different paths, meet and end in one purpose—and that purpose the cure.

> "As many arrows, loosed several ways,
> Fly to one mark."

The following extract contains a general account of the course of treatment which he would inculcate:—

"When from the pulse I have considered venesection necessary to bring down the circulation, the loss of between twelve and sixteen ounces of blood has generally been enough to answer the purpose in view; and the venesection has seldom been repeated."

"The opium, and calomel, and purgatives I have been accustomed to give in combinations thus:—With the calomel administered at night, according to its quantity, I have united more or less of opium. To ten grains of calomel I have added one grain of opium; or to five of calomel I have added half a grain, continuing to give them together in the same proportion, night after night, as long as they are needed. Then, on each succeeding day, when a large purgation of the bowels has been duly obtained, I have still given the opium alone, or with saline draughts, in doses of half or one-third of a grain, every five or six hours. And thus, with a larger quantity at night, and the smaller quantities during the day, about two grains of opium have been commonly taken in the course of twenty-four hours."

Here, then, the vascular system, and the nervous system, and the abdominal viscera are all at the same time made to feel sensibly the impression of the remedy, but none of them is subdued by it. And with blood-letting, and opium, and calomel with purgatives are all made confederate for the cure of the disease, none of them is given in excess."

"Now, I do not pretend to say, that this is just the measure, and just the relative proportion in which these several remedies need always to be employed for the cure of acute rheumatism. There are circumstances which would require them to be varied. But apart from the patient, they cannot be represented intelligibly. As of venesection, so of these other remedies, after the propriety of their use is already understood, the skill of using them remains to be learnt; the skill which sees when to give a little more, and when a little less, oftener or seldomer—when to bear heavily or lightly on the blood-vessels, when heavily or lightly on the nerves—and when to obtain larger or smaller purgation of the abdominal viscera. This is the skill which cures diseases and saves lives. And no man ever had it, who did not obtain it from his own self-teaching amid the emergencies of actual practice."

With respect to colchicum, a remedy which by many is so highly praised in the treatment of this disease, we will quote his remarks at some length:—

Colchicum, single-handed, cannot (I think) be safely trusted for the cure of acute rheumatism in the severe cases, but it can in the milder ones ; and I have so trusted it: yet I do not recommend the practice. Colchicum given alone has been slow, even in these milder cases of making its curative impressions. Many days have generally elapsed before it has produced any abatement of swelling and of pain, of vascular action and of fever ; and then, not until it has *began to purge smartly and even painfully*. Now these are hardly satisfactory conditions upon which to obtain the remedial effects of colchicum. For, to purge by colchicum is to make it act as it does in its first degree of poisoning."

"Finding, then, that in the milder cases I had no fair chance of obtaining from it the virtue of a remedy without running some hazard of it as a poison, it was much too dangerous an experiment in my eyes to commit the treatment of acute rheumatism to it mainly or entirely in the severe cases. For now it must be pressed nearer and nearer to the verge of poisoning in order to bring it at all within the capacity of curing."

"But in all cases of acute rheumatism, both mild and severe, the practice prevails of giving colchicum, not alone, but has an auxiliary to other remedies. To bleeding and opium and calomel and purgatives, given in the manner specified,many would add colchicum. They would prescribe a grain of the acetuous extract, or fifteen or twenty minims of the wine twice or thrice a day, some considering it to act sedatively and as a special auxiliary to opium, and some specifically and with the force of an antidote, as it does in gout."

"I, too, use colchicum in acute rheumatism, but not after this manner. I reserve it for special emergencies; and then I employ it with a trust and confidence which I have in no other remedy."

"When by venesection and by opium and calomel with purgatives, excess of vascular action, and fever and pain and swelling are abated, yet none of them are entirely abolished, but all still linger; or when pain and swelling do not subside at all in proportion to the abatement of vascular action and fever, which are considerably reduced, then I invoke the aid of colchicum, and give twenty or five-and-twenty minims of the wine of the seeds or the root, twice or thrice a day, and I often find the disease proceed uninterrupted-to its cure."

"Again, when by the same ordinary course of treatment fever, pain, and swelling have been made to cease entirely, and have suddenly and unexpectedly returned, then I invoke the aid of colchicum, and give it in the same way; and a few doses are commonly enough to dissipate the returning disease, and restore the conditions of health. This is a pure case of relapse. The relapse, however, very seldom reaches the severity of the orginal attack.

Now on all such emergencies I have been accustomed to administer the remedy quite alone, uncombined with either alkali or opiate, so that the benefit which has resulted has been without question exclusively due to it; and not only exclusively due to it, but due to it purely as colchicum in virtue of that mode of action (whatever it be) which specifically belongs to it. For the cure has followed suddenly, and not waited for any intermediate operation of the remedy upon the stomach and bowels."

It does not appear from the foregoing extract that Dr. L. has been in the habit of administering the colchicum with that freedom which, in the hands of some other practioners, has proved in a high degree successful. That its efficacy is by some over-estimated, and that it should be given with discriminating caution, is no doubt true; but we are disposed to think that it posseses more value as a remedy in rheumatism than is accorded by the faint praise which Dr. L., bestows upon it.

So much for the treatment of rheumatism considered as a febrile disease complicated with inflammation of the joints, in which aspect alone, until of late years, it has been regarded. We come now to consider the *preventive treatment of rheumatic endocarditis and pericarditis.* These complications almost uniformly are preceded by the affection of joints for a greater or less period. They seldom appear at the commencement of the rheumatic attack. Cases, however, do occur occasionally, in which both take place consentaneously, and, indeed, Dr. L., has met with a few cases in which the inflammation of the heart took precedence of the arthritic affection. But in the vast majority of cases, if we attend them from the beginning, there is a certain period, a few days, or, at least hours, in which the complications under consideration are not yet developed. How can we pursue any special treatment to avert the occurrence? Dr. L., does not lay down any *special* means for this end. He thinks that the treatment, which, aside from these complications, is most judicious, will be most likely to be effectual as preventives. But he enjoins the importance of watching for the earliest indications of heart disease so as to determine the moment of its inception. The attrition or bellows' murmurs, or both, will furnish, of course, unequivocal evidences that this organ has become implicated; but even before the development of physical signs, according to Dr. L.'s experience, præcordial pain, excessive impulse, and irregular actions, alone or combined, render the occurrence of disease in the organ so certain, that it should at once influence our treatment. When these symptoms are prevented we may expect that they will shortly be followed by auscultatory, signs which will dispel all doubt; but we should not wait for the latter before resorting to measures which have reference to the condition of the organ in question. We quote passages which contains the pith of the practical precepts which Dr. L., inculcates.

"Bleeding, mercury, opium, the very remedies you used in acute rheumatism, are (I say) still your main reliance, when inflammation attacks the heart; but bleeding in different modes and measures, mercury directed to a totally different purpose, and opium given with more than one single intention."

"As soon as inflammation is known or suspected to have reached the heart, mercury must be given without delay. Or should mercury be already in use, as a remedy for acute rheumatism, with the intent of obtaining large evacuations from the bowels, it must at once have a new direction given to it. The irritation it has produced within the abdomen must by all means be pacified, and its constitutional impression must now alone be thought of, — that impression, of which salivation is the best evidence, of which (as far as we know) it alone, of all remedial substance, is properly and exclusively capable, and which, under favorable circumstances, is largely counteractive of inflammation."

"But inasmuch as that impression of mercury, or salvation, which is the best evidence of it, cannot be commanded within any given time, you must be content to administer it in the manner calculated for this purpose, and wait the event. For no judgment can be formed of its curative effect upon the disease until salivation arrives. And it may arrive in a day or two, or not until after several days, or after a week, or several weeks, or it may not arrive at all. You must give mercury, then, and wait until salivation come to tell you what it has done for the disease, or until it is fair to conclude there will be no salivation."

"With respect to venesection, if in acute rheumatism, any of those symptoms referable to the heart are present, which have been already mentioned, auscultatory or non-auscultatory, and especially if they have arisen under your own observation, or, though not under your own observation, if they be now present, and you have reason to believe that they have *recently* arisen, then should the pulse be found to have even a notable degree of that hardness which is deemed inflammatory, blood must be taken from the arm. Should there be any doubt about venesection, any misgiving whether the inflammatory hardness of the pulse is enough to require it, let it be employed nevertheless. There is greater hazard in omitting it wrongfully than in practising it wrongfully."

Local depletion by leeching or cupping is also recommended. Dr. L., prefers the cups, which he thinks are best applied between the left scapula and the vertebral column.

Opium will be useful to abate pain, and calm the nervous system as in cases of rheumatism without these complications, but more especially with them in order to restrain the cathartic operation of calomel, and promote a speedly salivation. In some cases a sudden agony will seize the heart, and, to use the language of the author, "the patient will feel and look as if he had received his death blow." It resembles spasms of the heart, and is, in reality, an attack of angina pectoris. Opium given in large doses is the proper and only remedy for this accident.

Finally, the author enforces the necessity of discriminating between the progressive inflammatory processes, and the evidences of disorder occasioned by the mechanical obstacles which result from the permanent effects of these processes upon the valves of the heart. It is only during the period occupied by the former that active treatment is called for, the latter are irremediable, and to continue the treatment because the signs of unsoundness of heart are present, would be useless and injurious.

Dr. L., devotes two lectures to the discussion of mercury as a remedy for inflammations in general, and afterwards resumes the subject of its special applicability to rheumatic pericarditis and endocarditis. He does not, however, present views which are new or original. He contends, in the first place, for the efficacy of this remedy in counteracting inflammatory action, or for (as he chooses to call it) its *anti-phlogistic* influence. His explanation of its *modus operandi* is, that "it constrains the morbid energy of the blood-vessels, and counteracts the powers by which the inflammation is carried on." This is a mere verbal explanation, conveying no definite ideas of the nature of the influence exerted. But this is all that in the present state of knowledge was to have been expected from an attempt to supply a rationale of its operation. He impresses the point, which is by no means peculiar to his instructions, that its efficacy is especially manifested in those forms and seats of inflammation in which the effusion of plastic lymph is the most characteristic event. Hence, in inflammation of the serous tissues it is particularly applicable, and in those rare varieties of mucus inflammations, which are of a diptheritic character, as croup. Hence, also, it is more especially adapted to those conditions of the system which are favorable to lymphatic euxdations, and is less suited to certain states, as scrofula, in which local inflammations are less likely to assume that character. In short, he considers, as do most modern practical writers, that in the anti-plastic properties of the remedy, its virtue, as an anti-phlogistic, chiefly resides. He illustrates this remarkable property by the instance of *iritis*, which is so often adduced for the same purpose, and no doubt furnishes a beautiful exemplification of its remarkable agency in some cases.

But in addition to the properties just refered to, mercury aids the restorative processes—processes instituted to restore parts from the effects of inflammation. It serves to remove the morbid condition in which parts, which have suffered acute inflammation, are left, and it causes the absorption of the morbid products of inflammatory action. This he distinguishes as the *reparatory* effects of the remedy.

Both these effects of mercury render it appropriate, and, in the view of Dr. L., peculiarly important in inflammatory affections of the heart. He advocates in the warmest terms its utility from the results of his own experience. The same view is taken by Dr. Hope, and, we presume, by most English as well as American writers. The French practitioners, however, it appears, following Bouillaud, do not adopt the mercurial practice, but trust to depletion alone. Dr. L., bases the evidence of the merits of the mercurial plan of treatment upon a comparison of the success which respectively obtains in the two countries. We give his own words on this topic:—

"These, moreover, are the cases in which foreign and English practice in the management of pericarditis may be fairly brought into comparison, and in which it may be seen where and how the one so often fails, and the other is so often successful."

"In foreign practice, no mercury is used from first to last, but all the power of common antiphlogistic remedies is brought to bear upon the disease; and thus its symptoms are mitigated or subdued : yet they return again and again, and are again and again mitigated or subdued. And so the patients are kept alive for a week or ten days, and then they die in the great majority of cases."

"In English practice, mercury is given from first to last. But it is for a long time as if it were not given at all, for it produces no sensible effect. Common antiphlogistic remedies, however, are able again and again to mitigate and subdue symptoms; and so at the end of a week or ten days the patients are still alive. Yet they are ready to die ; but in the great majority of cases they do not die. Salivation arrives late, and seems to save them."

We are, of course, not prepared to say whether too much is assumed, on the one hand, in the foregoing comparison; or, on the other hand, whether injustice is done to the French practitioners. It is but fair to remind the reader that the writer is an Englishman, and that since he does not furnish statistics of cases treated on both sides of the British channel, his statement is to be received as *exparte* testimony. We do not say this in disparagement of the value which should be attached to Dr. Latham's extensive experience, nor in distrust of the alledged efficacy of the remedy in these and other inflammatory affections. Every practitioner has abundant occasion to witness remarkably curative effects from the administration of mercurials: but like all other active remedial agents, this is potent for evil, as well as benefit, unless directed with judicious discrimination, and regulated with prudent caution. The indiscriminate and excessive use of this mineral, in our view, has been productive of disasters, which, perhaps, have more than balanced its advantages; and we should have been better satisfied with Dr. Latham's lectures on the subject, if he had presented more freely and emphatically, the dangers to be apprehended from its injudicious administration, in connection with the considerations which enforce its usefulness and importance.

We will here leave the work for the present, intending to resume our analytical review in the next, or a subsequent number.

